# Novel Presentation of Reticulate Acropigmentation of Kitamura With Bilateral Clinodactyly

**DOI:** 10.7759/cureus.26894

**Published:** 2022-07-15

**Authors:** Ariel Kidron, Skyler Coetzee, Daren Fomin

**Affiliations:** 1 Emergency Medicine, Nova Southeastern University Dr. Kiran C. Patel College of Osteopathic Medicine, Fort Lauderdale, USA; 2 Medical School, Nova Southeastern University Dr. Kiran C. Patel College of Allopathic Medicine, Davie, USA; 3 Dermatology, Irwin Army Community Hospital, Fort Riley, USA

**Keywords:** immuno-histochemical, reticulate pigmentary disorder, dyschromatoses, reticulate hyperpigmentation, cutaneous hyperpigmentation

## Abstract

Reticulate acropigmentation of Kitamura (RAPK) is a rare genetic hyperpigmentation disorder that is a member of the dyschromatoses characterized by hyperpigmented macules or papules that may interrupt the dermatoglyphics with extra-dermatological manifestations. We present a case of a 29-year-old black male who presented with hyperpigmented atrophic macules both on the extremities and genitals, as well as bilateral clinodactyly of the 5th fingers and inferior gingival hyperplasia with teeth crowding, to draw attention to the novel extra dermatological manifestations of RAPK and the differential diagnosis of cutaneous hyperpigmented lesions.

## Introduction

Reticulate acropigmentation of Kitamura (RAPK) is a rare genodermatosis characterized by an autosomal dominant inheritance pattern and multisystem involvement with predominance in females with Japanese heritage. This pigmentary anomaly first appears during childhood and is differentiated from other hyperpigmentation disorders by having sharp and demarcated reticulate hyperpigmented macules encompassing the dorsa of the forearms and hands combined with fine pitting and interruptions in the dermatoglyphics and palmoplantar pits [1,2]. RAPK may be confused with the reticulate acropigmentation of Dohi due to some overlapping characteristics and this duality may present challenges to clinicians in successful diagnosis and treatment [1]. RAPK has a worldwide prevalence of less than one in a million and few cases have been reported in the Americas with even fewer detailing sequelae of bony and gum abnormalities [3-5]. Herein we present a novel case of a 29-year-old black male who presented with hyperpigmented atrophic macules on his hands, feet, and genitals with bilateral clinodactyly of the 5th fingers and inferior gingival hyperplasia with teeth crowding. To our knowledge, this is the first case of reticulate acropigmentation of Kitamura with bony abnormalities in the hands documented in the Americas, thus presenting a unique kaleidoscope of dermatological and extracutaneous presentations to draw attention to the unique manifestations of RAPK and the differential diagnosis of hyperpigmentation disorders.

## Case presentation

**Figure 1:**
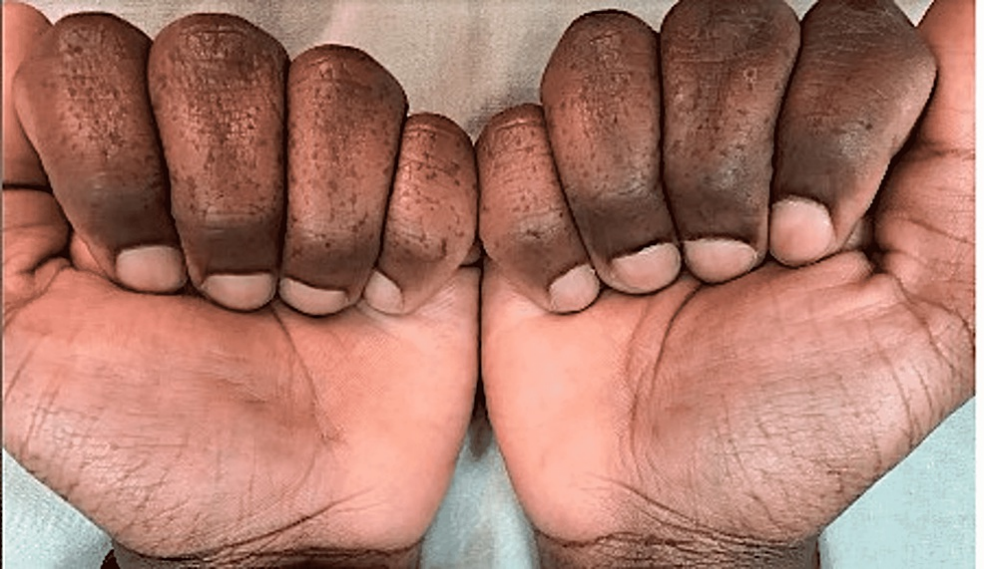
Reticulate acropigmentation of Kitamura depicting initial lesions.

A 29-year-old male (Fitzpatrick Skin Type V) with unremarkable past medical history originally presented to the clinic with complaints of a nevus on the foot which was determined to be benign. This initial presentation prompted the incidental finding of hyperpigmentation on the hands and feet. Upon physical exam, stellate hyperpigmented atrophic macules could be visualized. The patient reported the lesions to be present since childhood, and the lesions first appeared on the hands and feet and have been stable since their initial appearance (Figure 1).

**Figure 2:**
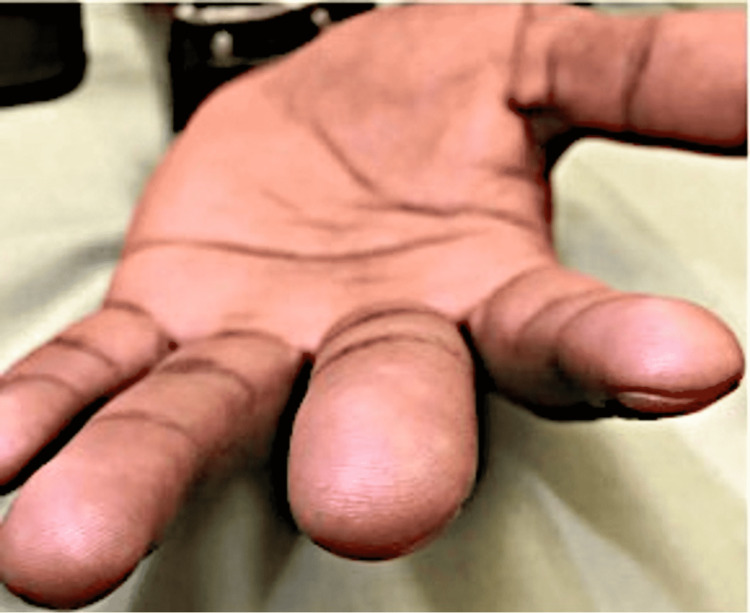
Reticulate acropigmentation of Kitamura showing palmar pitting and breaks in the dermatoglyphs.

Family history revealed a sister with similar skin findings on the hands but without a known diagnosis. The patient endorsed similar lesions on his genitals and denied hypopigmentation, associated pruritus, pain, or bleeding from the lesions. Cutaneous examination showed the hyperpigmented lesions contained palmar pitting and breaks in the dermatoglyphs (Figure 2) in addition to bilateral clinodactyly of the 5th fingers as well as inferior gingival hyperplasia with teeth crowding as evidenced by thickening of the gum tissue and competition of the teeth for growth space raised suspicion for reticulate acropigmentation of Kitamura.

**Figure 3:**
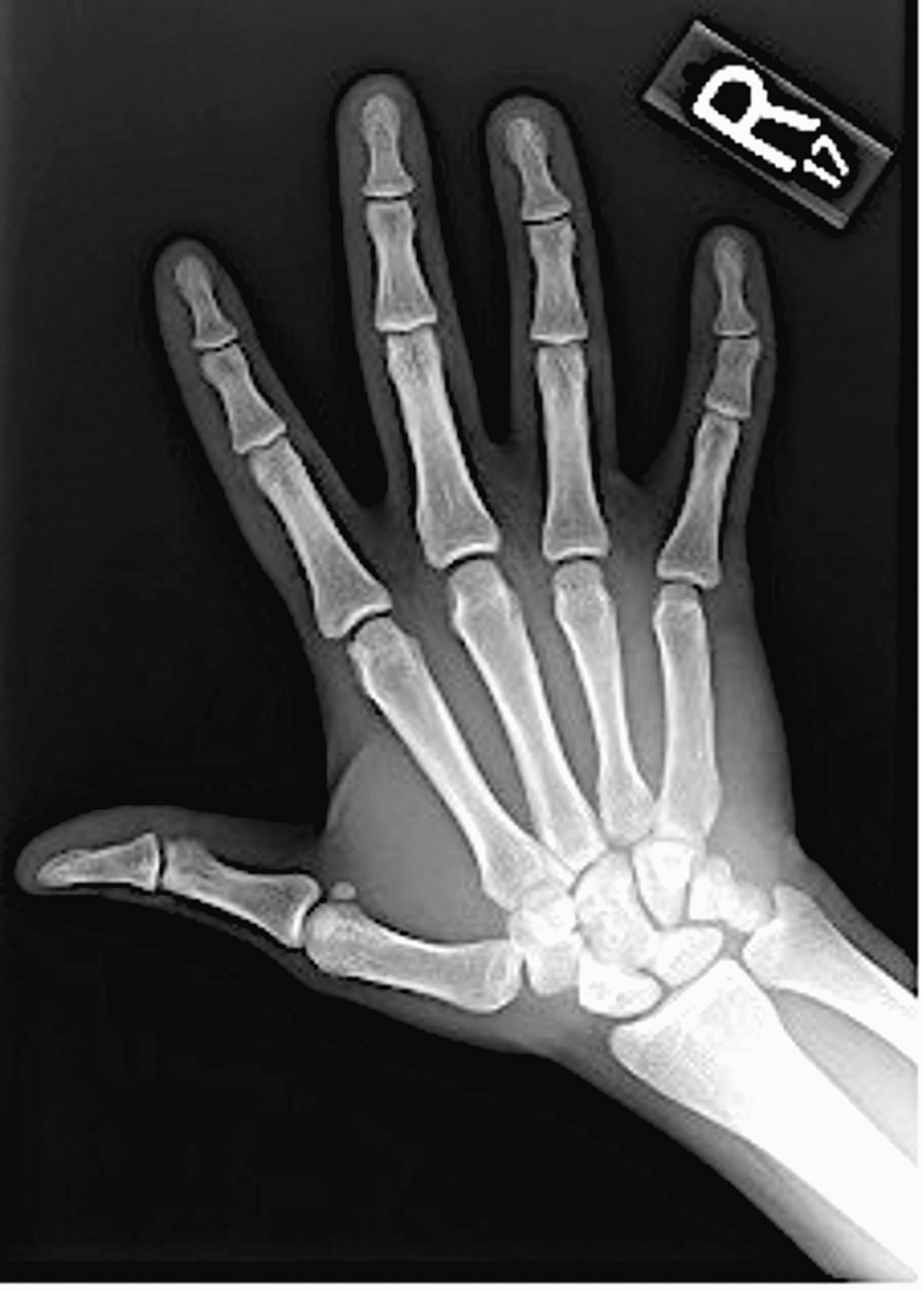
Reticulate acropigmentation of Kitamura presenting with clinodactyly of the right hand on plain film.

**Figure 4:**
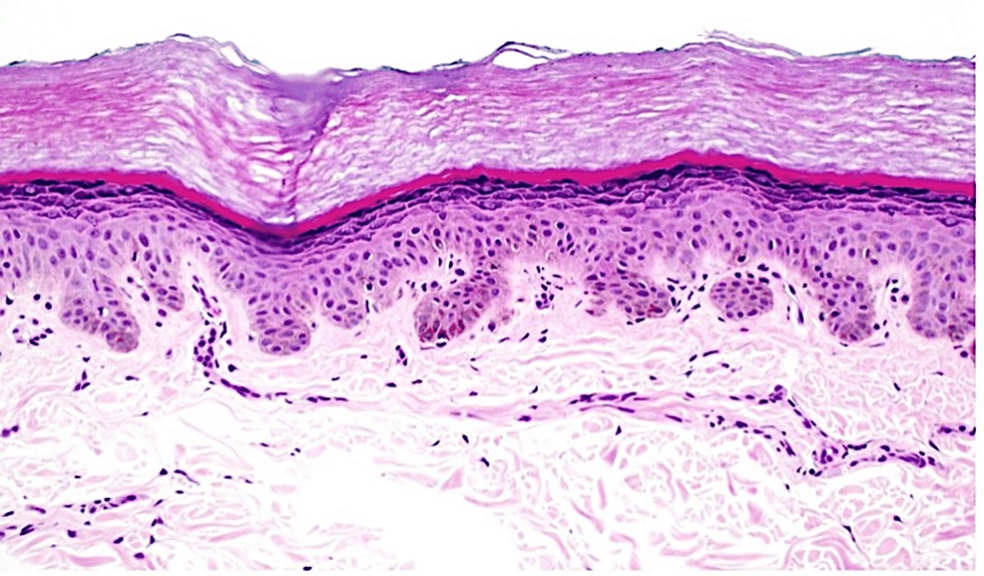
Reticulate acropigmentation of Kitamura histology (H+E stain, x40 Magnification) The image depicts epidermal atrophy with elongation of the rete ridges and basilar hyperpigmentation

X-ray imaging of the hands was ordered to better characterize the bony changes and displayed mild congenital foreshortening of the fifth finger middle phalanges bilaterally with the remodeling of the distal heads having slight shallow and radially oriented surface articulation of the head of the middle phalanges causing slight radial angulation of the distal phalanges bilaterally and consistent with the extra dermatological presentation of RAPK (Figure 3). Skin biopsy of the dorsal hand supported the diagnosis of reticulate acropigmentation of Kitamura by revealing a focus of epidermal atrophy with elongation of the rete ridges and basilar hyperpigmentation (Figure 4).

## Discussion

The classical clinical progression of RAPK begins in childhood with the development of atrophic hyperpigmented papules or macules resembling lentigines on the dorsal palmar-plantar surfaces that coalesce into a reticular, net-like pattern. These lesions may further darken and spread with sunlight exacerbating progression. Other cutaneous manifestations include pitting of the palmar-plantar surfaces and discontinuity of dermatoglyphs [1]. Notably, hypopigmented lesions are absent in RAPK, differentiating it from the inherited dyschromatoses which feature both hypo and hyperpigmented lesions [6,7]. Our patient’s case is unique in that he is male, while RAPK is known to be predominant in females. Furthermore, he presented with extra-cutaneous manifestations including gingival hyperplasia and shortening of the fourth and fifth phalanges of the hand. The only published case of bony abnormalities associated with RAPK involved the absence of the terminal phalanges of the second, third, and fourth toes [5]. The patient’s sister was also reported to have similar cutaneous findings on her hands consistent with the autosomal dominant inheritance pattern of RAPK. 

A mutation resulting in the loss of function of ADAM10, a gene encoding a notch signaling pathway activator metalloproteinase, is key in the pathogenesis of RAPK. The gene typically works in the ectodomain shedding of membrane proteins with L1-CAM, CD44, E-cadherin, N-cadherin, IL-6 receptor and CD30 acting as substrates [1]. Typical findings on histopathological evaluation of lesions show club-like elongation of the rete ridges, epidermal atrophy, hypermelanosis, and excess melanocytes within the basal layer. Only a few inflammatory cell infiltrates and no incontinentia pigmenti are seen in the dermis [8-10].

The differential diagnosis for RAPK includes other inherited reticulate pigmentary disorders and pigmented skin lesions such as reticulate acropigmentation of Dohi (RAPD) [6,10]. These conditions are often differentiated only by subtle clinical differences in presentation. For example, in RAPK there are no hypopigmented macules and histologically there is epidermal atrophy and an increase in melanocytes while in RAPD there is acantholysis in the suprabasal epidermal layers [6,10]. Several studies report the coexistence of such disorders in the same patient, suggesting that they may even represent a spectrum of reticulate pigmentary dermatosis rather than distinct pathologies [7]. Further elucidation of the genetic underpinnings of these rare genodermatoses is necessary to better characterize this relationship. Effective treatment options for RAPK are still under investigation. Both topical and systemic retinoids have had minimal lasting effectiveness in resolving lesions, and azelaic acid is another topical option with potential. Laser therapy traditionally used for other pigmentary conditions, such as the Erbium YAG laser, is another treatment modality that may improve the appearance of skin lesions [2,10].

## Conclusions

Reticulate acropigmentation of Kitamura is a member of an extremely rare family of skin dyschromatoses that may arise during the second and third decades of life. RAPK shares overlapping features with other hyperpigmentation disorders in addition to having extra-dermatological manifestations which make the initial diagnosis challenging. Thus, it is important for clinicians to be aware of the nature, pathology, and treatment options when encountering RAPK as well as the distress the pathology may have on the patient. While the management of RAPK may prove daunting and the efficacy of treatment options is uncertain, the continued research and experimentation with novel immunotherapy may pave the way for improved outcomes. 
