# Correction: Novel Presentation of Reticulate Acropigmentation of Kitamura With Bilateral Clinodactyly

**DOI:** 10.7759/cureus.c71

**Published:** 2022-08-22

**Authors:** Ariel Kidron, Skyler Coetzee, Daren Fomin

**Affiliations:** 1 Emergency Medicine, Nova Southeastern University Dr. Kiran C. Patel College of Osteopathic Medicine, Fort Lauderdale, USA; 2 Medical School, Nova Southeastern University Dr. Kiran C. Patel College of Allopathic Medicine, Davie, USA; 3 Dermatology, Irwin Army Community Hospital, Fort Riley, USA

This article has been corrected due to the erroneous omission of second author, Skyler Coetzee, by the submitting author. Skyler Coetzee is now correctly listed as the second author. The authors sincerely regret the error.

